# A comprehensive investigation on the receptor BSG expression reveals the potential risk of healthy individuals and cancer patients to 2019-nCoV infection

**DOI:** 10.18632/aging.205655

**Published:** 2024-03-13

**Authors:** Yongbiao Huang, Haiting Zhou, Yuan Wang, Lingyan Xiao, Wan Qin, Long Li

**Affiliations:** 1 Department of Oncology, Tongji Hospital, Huazhong University of Science and Technology, Wuhan, China

**Keywords:** SARS-CoV-2, COVID-19, BSG, cancer, susceptibility

## Abstract

Background: Coronavirus disease-2019 (COVID-19) pandemic is caused by severe acute respiratory syndrome coronavirus 2 (SARS-CoV-2), a newly emerging coronavirus. BSG (basigin) is involved in the tumorigenesis of multiple tumors and recently emerged as a novel viral entry receptor for SARS-CoV-2. However, its expression profile in normal individuals and cancer patients are still unclear.

Methods: We performed a comprehensive analysis of the expression and distribution of BSG in normal tissues, tumor tissues, and cell lines via bioinformatics analysis and experimental verification. In addition, we investigated the expression of BSG and its isoforms in multiple malignancies and adjacent normal tissues, and explored the prognostic values across pan-cancers. Finally, we conducted function analysis for co-expressed genes with BSG.

Results: We found BSG was highly conserved in different species, and was ubiquitously expressed in almost all normal tissues and significantly increased in some types of cancer tissues. Moreover, BSG at mRNA expression level was higher than ACE2 in normal lung tissues, and lung cancer tissues. High expression of BSG indicated shorter overall survival (OS) in multiple tumors. The Gene Ontology (GO) and Kyoto Encyclopedia of Genes and Genomes (KEGG) pathway analyses indicated that BSG is mostly enriched in genes for mitochondria electron transport, oxidoreduction-driven active transmembrane transporter activity, mitochondrial inner membrane, oxidative phosphorylation, and genes involving COVID-19.

Conclusions: Our present work emphasized the value of targeting BSG in the treatment of COVID-19 and cancer, and also provided several novel insights for understanding the SARS-CoV-2 pandemic.

## INTRODUCTION

Coronavirus disease 2019 (COVID-19), a novel respiratory tract infection caused by severe acute respiratory syndrome coronavirus (SARS-COV-2), initially broke out in December 2019 [1–3]. As far, the global confirmed cases are 532,355,835, and the global death cases are 6,300,060 based on the report from the Center for Systems Science and Engineering (CSSE) (https://coronavirus.jhu.edu/) (June 2022) [4]. SARS-CoV-2 is an enveloped, single-strand RNA virus of the genus Betacoronavirus [5]. Patients with COVID-19 presented lung computed tomography (CT) abnormalities, along with mild flu-like symptoms (e.g., headache, fatigue, cough, nasal congestion and fever [6]. The pathological characteristics of COVID-19 patients are very similar to those of SARS and MERS patients [7]. Middle-aged and older patients with comorbidities are prone to respiratory failure resulting in poor clinical outcome [8].

SARS-CoV-2 (COVID-19) is highly homologous to SARS-CoV responsible for an outbreak in 2003 [9]. SARS-CoV-2 shares 79.5% genetic characteristics with SARS-CoV [5]. Both SARS-CoV-2 and SARS-CoV enter into cells by spike glycoproteins, which binds membrane protein angiotensin-converting enzyme II (ACE2) within the host [10–12]. Nevertheless, recent studies have confirmed that the binding affinity of SARS-CoV-2-related spike protein to ACE2 is 10–20 folds stronger than that of SARS-CoV to ACE2 [13, 14]. This enhancement affinity with ACE2 is associated with the higher transmissibility of SARS-CoV-2 compared to SARS-CoV. ACE2 is expressed in gastrointestinal tract, kidney and lung, however, its expression level is very low, especially in lung [15]. Given the high transmissibility of SARS-CoV-2, we hypothesized that there might be other underlying receptors to promote its infection.

BSG (basigin), also known as CD147 or EMMPRIN, is a plasma membrane protein of immunoglobulin superfamily [16]. BSG is widely expressed in epithelial and immune cells and served a variety of vital functions, including inflammatory, immune responses, tumor progression, bacterial and viral infections [17–20]. CD147 has three N-glycosylation sites, and high mannose-type and complex glycans may bind to them [21]. The spike protein of SARS-CoV-2 is highly glycosylated, which increases the chance of binding to cells [22]. As a target related to SARS-CoV-2 spike protein, BSG can enable SARS-CoV-2 to enter host cells through endocytosis. It even activates the MAPK pathway axis and induces cytokine storms via the spike protein/BSG/CyPA signal [23]. Thus, BSG is a key factor in COVID-19 infection and progression and can serve as a new target for effective treatment of COVID-19. In addition, BSG has numerous interacting partners, including cyclophilin A (CyPA), a member of the immunophilin family, is also important in viral infections [24]. The ability of viruses to invade into target cells relies on the interactions between BSG and CyPA [18]. The SARS-CoV nucleocapsid protein binds to CyPA, and further recognize BSG receptors on the cell surface of host [25]. It was reported that SARS-CoV-2 spike protein interacted with the host cell receptor BSG as a novel entry route. The regulation of BSG level influenced the ability of virus to invade into host. In addition, BSG takes part in SARS-CoV-2 invasion into immune cells, which do not express ACE2 [26].

Cytokine storm occurred in the majority of severe COVID-19 cases, which featured as increased serum cytokine and chemokine levels (e.g., IFN-γ, and TNF-α, CCL2, CXCL10, IL-1, IL-6) [27]. The cytokine storm is followed by acute respiratory distress syndrome (ARDS) and multiple organ failure (MOF), which leads to death in severe COVID-19 patients [28]. BSG was reported to participate in the cytokine storm via modulation of CyPA expression, and anti-BSG antibody potently reduce the infection and cytokine storm of SARS-CoV-2 [29].

Patients with malignancies are more vulnerable to SARS-CoV-2 infection [30], more importantly, definitively susceptible to develop severe complications [31]. The mortality rate in patients with malignancies has been reported to be 25–30% [32]. In the present study, we conducted a comprehensive analysis on expression level of a novel SARS-CoV-2 receptor BSG in healthy tissues and pan-cancers. This might reveal the underlying implications of BSG in terms of SARS-CoV-2 infection.

## MATERIALS AND METHODS

### Expression analysis of BSG

The mRNA and protein expression of BSG in normal and tumor tissues were analyzed using the Human Protein Atlas (HPA) database (https://www.proteinatlas.org/), which composes of six modules: tissue atlas, brain atlas, metabolic atlas, blood atlas, pathology atlas, and cell atlas [33–35]. The mRNA expression of BSG for multiple immune cells and distinct cell lines, the immunohistochemistry data of BSG in lung cancer and normal lung tissues were analyzed by this database. The transcript data of BSG across pan-cancers and adjacent tissues were obtained from GEPIA2 database, which is publicly accessible at http://gepia2.cancer-pku.cn/, including data from The Cancer Genome Atlas (TCGA) and Genotype Tissue Expression (GTEx) projects [36]. Data were accessed on May 2022.

### Isoform usage profiling and isoform structure of BSG

GEPIA2 generated violin plots to visualize the expression level (log2(TPM + 1)) of each isoform for BSG, and bar plots to show the isoform usage (from 0% to 100%) distribution across multiple cancer types. Meanwhile, GEPIA2 provided isoform protein domain map, which is used to show the structural differences among different isoforms. Data were accessed on May 2022.

### Mutation and homology analysis of BSG

“Mutation module” of TIMER2.0 (http://timer.cistrome.org/) was used to analyze the mutation frequency of BSG for pan-cancers. The results were visualized by a bar plot. Homology analysis of BSG was performed by the NCBI program (https://www.ncbi.nlm.nih.gov/homologene/1308) [37]. Data were accessed on May 2022.

### Survival analysis of BSG

GEPIA2 generated a survival heat map to present the prognostic impacts of BSG expression, with the hazard ratios in logarithmic scale (log10) for multiple cancer types. According to median expression of BSG, samples were divided into two groups, high BSG group and low BSG group. Then, GEPIA2 performed survival analyses based on Kaplan-Meier curves with log-rank test for OS and disease-free survival (DFS) [38]. Data were accessed on May 2022.

### Functional enrichment analysis

The top 100 co-expressed genes with BSG were identified using GEPIA2. Then, the Enrichr database (http://amp.pharm.mssm.edu/Enrichr/) was subjected to GO functional annotation and KEGG pathway enrichment analysis based on these co-expressed genes [39]. GO contains three categories: molecular function (MF); biological process (BP); and cellular component (CC). In addition, pathway enrichment analysis was also performed for COVID-19-related gene sets and virus perturbations from the Gene Expression Omnibus (GEO) [40, 41].

### Cell culture and reagents

A normal human lung epithelial cell line (BEAS-2B) and four lung cancer cell lines (H1299, PC-9, H1975, A549) were obtained from the American Type Culture Collection (ATCC, Manassas, VA, USA), and cultured in RPMI-1640 (HyClone, Logan, UT, USA) containing 10% fetal bovine serum (FBS, Gibco, Carlsbad, CA, USA).

### Clinical specimen collection

A total of five cases of human lung cancer tissues and the paired adjacent non-cancerous tissues were collected from Wuhan Tongji Hospital, approved by The Medical Ethics Committee of Tongji Hospital, Tongji Medical College, Huazhong University of Science and Technology.

### Immunohistochemistry (IHC)

Immunohistochemistry was conducted following the guidelines provided by the manufacturer. In brief, slides were deparaffinized, rehydrated, subjected to staining using the primary antibodies against BSG (1:100, A22443, Abclonal, USA).

### RNA extraction and qPCR

Total RNA was extracted using TRIzol reagent (Invitrogen, Carlsbad, CA, USA) according to the manufacturer’s protocol. cDNA was synthesized using HiScript II Q RT SuperMix (Vazyme Biotech, Nanjing, China) and used for quantitative polymerase chain reaction detection using ChamQ Universal SYBR qPCR Master Mix (Vazyme Biotech, Nanjing, China). The sequences of primers used are shown in Table 1.

**Table 1:** Sequences of primers used for qPCR.

**Gene**	**Forward or reverse**	**Sequences**
GAPDH	Forward	ACCCAGAAGACTGTGGATGG
	Reverse	TTCAGCTCAGGGATGACCTT
BSG	Forward	CAGAGTGAAGGCTGTGAAGTCG
	Reverse	TGCGAGGAACTCACGAAGAA

### Statistical analysis

The data of qPCR between two groups or multiple groups were analyzed with Student’s *t* test or ANOVA. The Kaplan-Meier (K-M) analysis was used to plot survival curves via the log-rank test. *P*-values < 0.05 were considered statistically significant.

## RESULTS

### The expression of BSG varies in different tissues or organs

**Figure 1:**
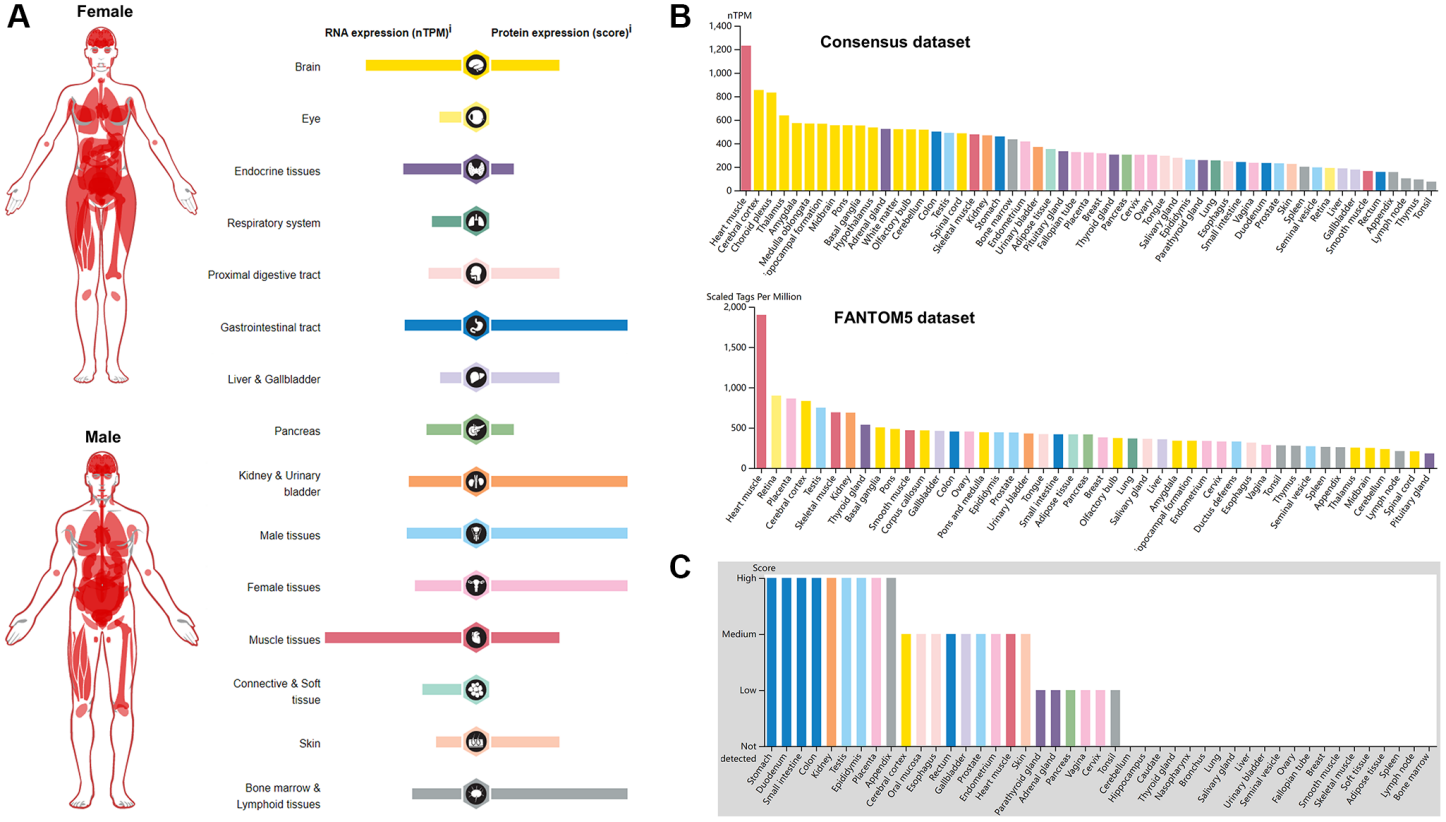
**Pattern of BSG expression in normal tissues.** (**A**) mRNA and protein expression profiles of BSG in different normal human tissues. (**B**) BSG mRNA expression in various tissues or organs from Consensus dataset and Fantom5 dataset. (**C**) BSG protein expression in various tissues or organs based on immunohistochemistry scores.

BSG is an essential entry receptor for SARS-CoV-2; Thus, we first analyzed the expression profile of BSG in normal tissues. The expression profile of BSG at the mRNA and protein level in different tissues were obtained from the HPA database. As shown in Figure 1A, BSG at mRNA expression is higher in muscle and brain tissues compared to that in other normal tissues. Eye and liver and gallbladder express the lowest BSG levels. Whereas, BSG at protein expression was different from its mRNA. BSG was expressed most highly in gastrointestinal tract, kidney and urinary bladder, generative organ and bone marrow and lymphoid tissues, with very little expression seen in eye, respiratory system and connective and soft tissue. Intriguingly, BSG exhibited a relatively low expression level in lung especially at the protein level, which was analogues to ACE2 expression pattern in lung [42]. We further validated the mRNA expression level of BSG in Consensus dataset and Fantom5 dataset. The heart muscle had the highest expression of BSG in both datasets. Cerebral cortex, choroid plexus and retina were reported to have the relative higher expression of BSG compared to other tissues (Figure 1B). The protein level of BSG was evaluated based on four score levels, high, medium, low, and not detected. There are nine organs at high score level (stomach, duodenum, small intestine, colon, kidney, testis, epididymis, placenta and appendix), nine organs at medium score level and six organs at low score level (parathyroid gland, adrenal gland, pancreas, vagina, cervix and tonsil). While, twenty-one tissues were not detected BSG protein (Figure 1C).

### Expression profile of BSG in plasma and blood cells

**Figure 2:**
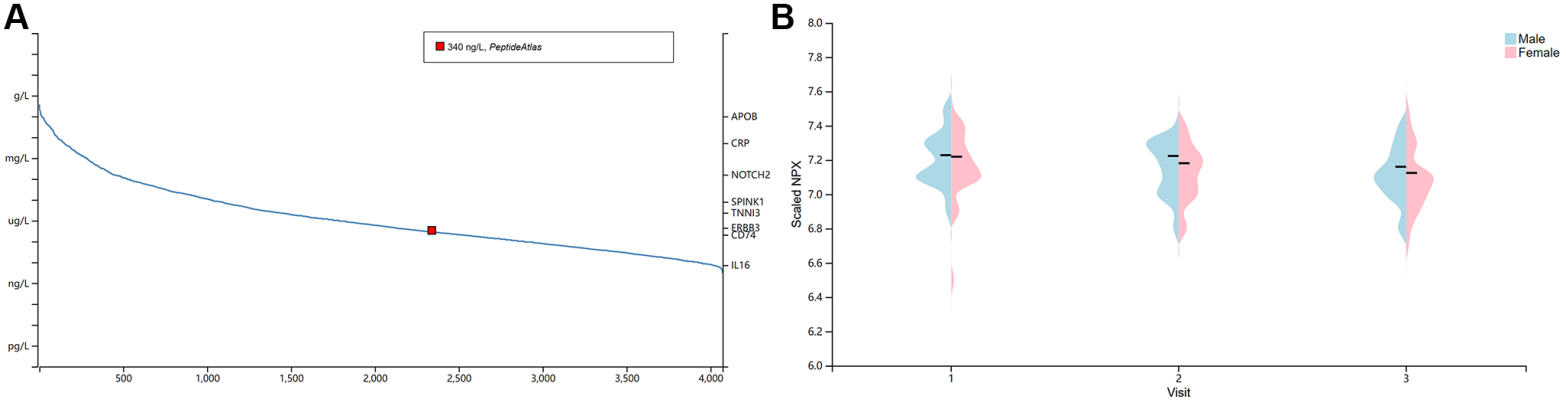
**The concentration of BSG in plasma.** (**A**) BSG protein concentration in plasma. Note: The red dots indicated BSG protein was detected in plasma with about 340 ng/L. (**B**) Differences between male and female in BSG protein concentration.

**Figure 3:**
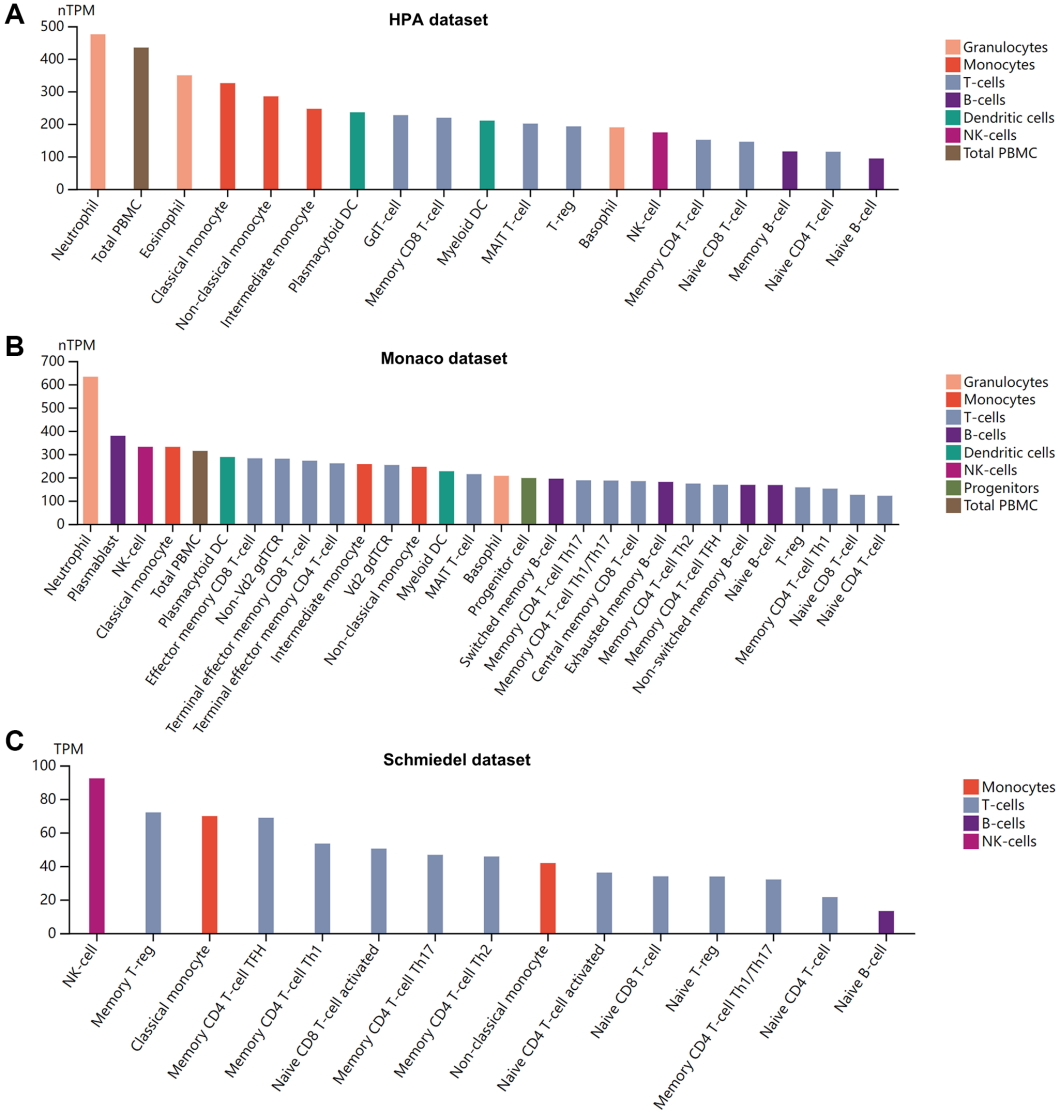
**Pattern of BSG expression in human blood cells.** (**A**) HPA dataset. (**B**) Monaco dataset. (**C**) Schmiedel dataset. X: blood cell types, Y: BSG expression value in transcripts per million (TPM).

High concentrations of cytokines were observed in the plasma of patients with severe COVID-19 [43]. The HPA database was used to quantify BSG protein concentrations in plasma using mass spectrometry and estimating from spectral counts. The results indicated that BSG at protein level was about 340 ng/l in plasma (Figure 2A). Previous studies have reported different susceptibility to COVID-19 between men and women [44]. Therefore, plasma protein was further measured using the proximity extension assay (PEA), with high specificity and sensitivity [45]. Surprisingly, plasma BSG protein was slightly higher in men than in women (Figure 2B). In addition, we investigated the BSG expression in immune cells based on 3 datasets, including HPA dataset, Monaco dataset and Schmiedel dataset. The results showed that BSG was enriched in Neutrophil and NK cells (Figure 3A–3C).

### Expression profile of BSG in malignancies

**Figure 4:**
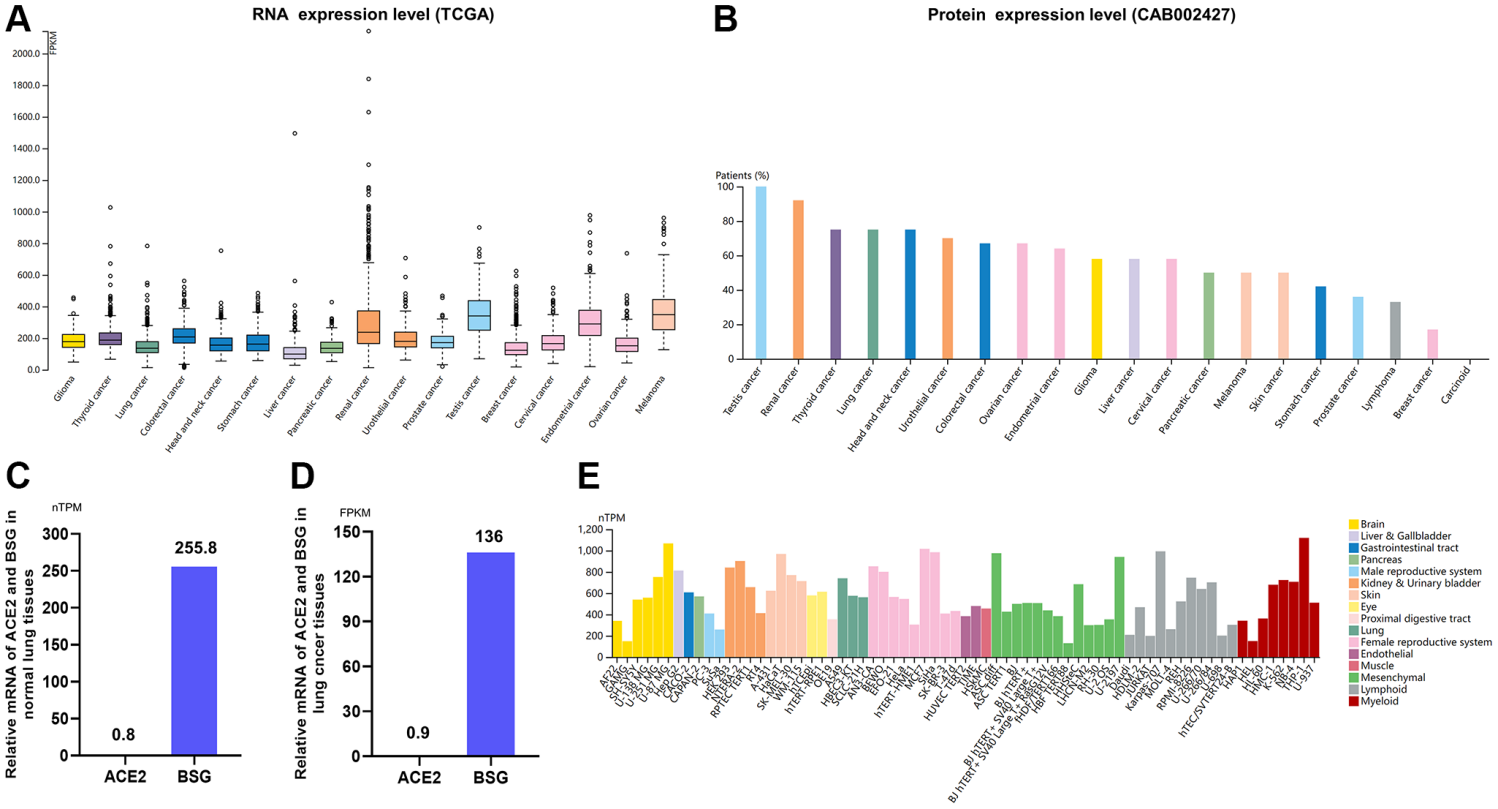
**Expression of BSG in tumor tissues and cancer cells.** (**A**) mRNA expression levels of BSG (TCGA) across multiple cancer types. (**B**) Protein expression levels of BSG (CAB002427) across multiple cancer types. (**C**) Relative mRNA expression levels of ACE2 and BSG in normal lung tissues. (**D**) Relative mRNA expression levels of ACE2 and BSG in lung cancer tissues. (**E**) mRNA expression levels of BSG in the multiple cancer and normal cells. The cancer types were color-coded according to which type of normal organ the cancer originates from. The cell lines were classified into 16 color-coded groups according to their organ to which it is from. Abbreviations: TPM: transcripts per million; FPKM, number of fragments per kilobase of exon per million reads.

Patients with cancer are more vulnerable to infect COVID-19 due to poor health conditions and immunosuppressive status induced by both the cancer and antitumor therapies [46, 47]. Expression profile of BSG at mRNA and protein level varied widely in different malignancies. The highest level of BSG mRNA was detected in testis cancer. While, the lowest level was observed in liver cancer (Figure 4A). At protein expression level, BSG was also the highest in testis cancer, which was consist with mRNA expression level. And BSG protein was hardly expressed in carcinoid (Figure 4B).

Then we compared the ACE2 mRNA and BSG mRNA expression in normal lung tissues and lung cancer tissues based on 994 samples from TCGA dataset. In terms of normal lung tissues, relative mRNA of ACE2 was 0.8 TPM and that of BSG was 255.8 TPM. And BSG mRNA expression level was 319.8 folds higher than ACE2 (Figure 4C). In terms of lung cancer tissues, relative mRNA of ACE2 was 0.9 FPKM and that of BSG was 136 FPKM. And BSG protein expression level was 151.1 folds higher than ACE2 (Figure 4D). In cancer cell lines, BSG mRNA expression was up-regulated when compared to normal cells (Figure 4E). These results indicated that BSG might be essential for the invasion of SARS-CoV-2 and COVID-19 pathogenesis in normal individuals and cancer patients.

### BSG isoform profile and mutation in pan-cancers

**Figure 5:**
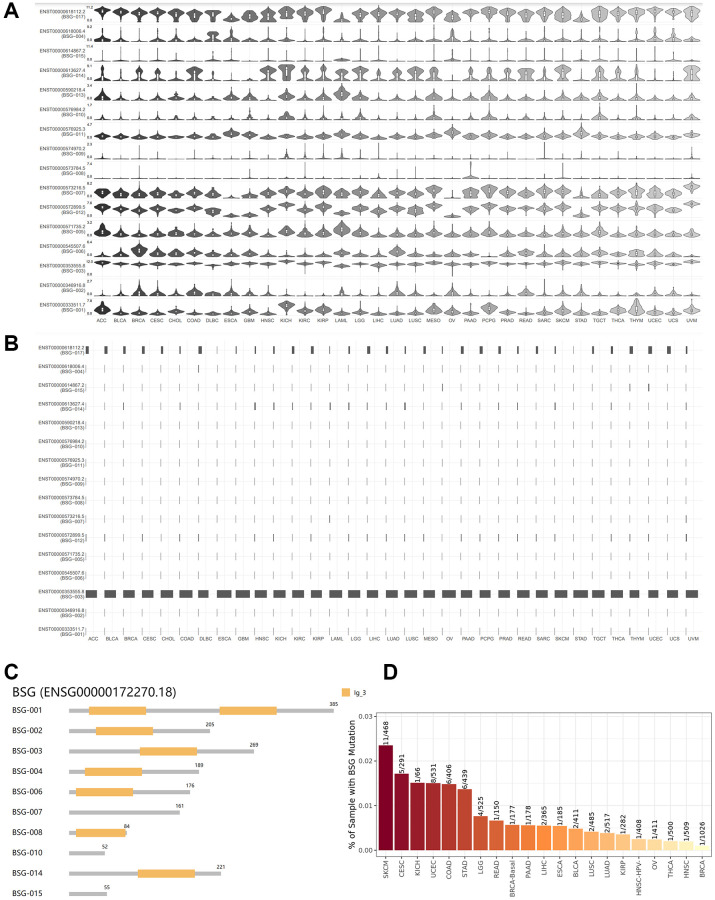
**Isoform usage and structures of BSG across multiple cancer types.** (**A**) Expression patterns of the BSG isoforms in different cancer types. X: cancer types, Y: isoforms of BSG. (**B**) The isoform usage of BSG in different cancer types. X: cancer types, Y: isoforms of BSG. (**C**) The structures of multiple BSG isoforms. Multiple isoforms and their protein domain structures are displayed in an interactive plot. (**D**) The mutant frequency of BSG in different cancer types.

Differential expression of ACE2 isoforms in respiratory epithelial cells has been reported due to distinct host susceptibility to SARS-CoV-2 infection [48]. Therefore, isoforms of other entry receptor for SARS-CoV-2 might exerted similar effects. As shown in Figure 5A, 5B, we observed 16 isoforms for BSG. And each isoform of BSG has distinct expression levels in different cancer types. BSG-003 (ENST00000353555.8) present the highest isoform usage distribution, followed by BSG−017 (ENST00000618112.2). Then we analyzed isoform structures of BSG. Six isoforms’ information was missing. They were BSG−005 (ENST00000571735.2), BSG−012 (ENST00000572899.5), BSG−009 (ENST00000574970.2), BSG−011 (ENST00000576925.3), BSG−013 (ENST00000590218.4), ENST00000618112.2 (BSG−017). Isoform BSG-001 contains two lg_3 domains. While isoforms BSG-002, BSG-003, BSG-004, BSG-006, BSG-008, and BSG-014 contain one lg_3 domain (Figure 5C). To sum up, isoform BSG-003 (ENST00000353555.8) might be essential for SARS-CoV-2 entry and tumor progression.

Tumors are often accompanied by multiple gene mutations, which leads to progression and relapse from therapy. Moreover, the mutation status of BSG in cancers may affect or correlate the expression of other entry receptors [49, 50]. Therefore, we conducted mutation analysis of the BSG gene in pan-cancers. The horizontal bar graph shows the BSG mutation frequency in Figure 5D. The highest BSG mutation frequency was observed in SKCM (11/468,2.35%), followed by CESC (5/291,1.71%), KICH (1/66,1.51%), UCEC (8/531,1.51%) and STAD (6/439,1.37%). The lowest BSG mutation frequency was observed in BRCA (1/1026, 0.10%).

### Pan-cancer analysis of BSG mRNA expression

**Figure 6:**
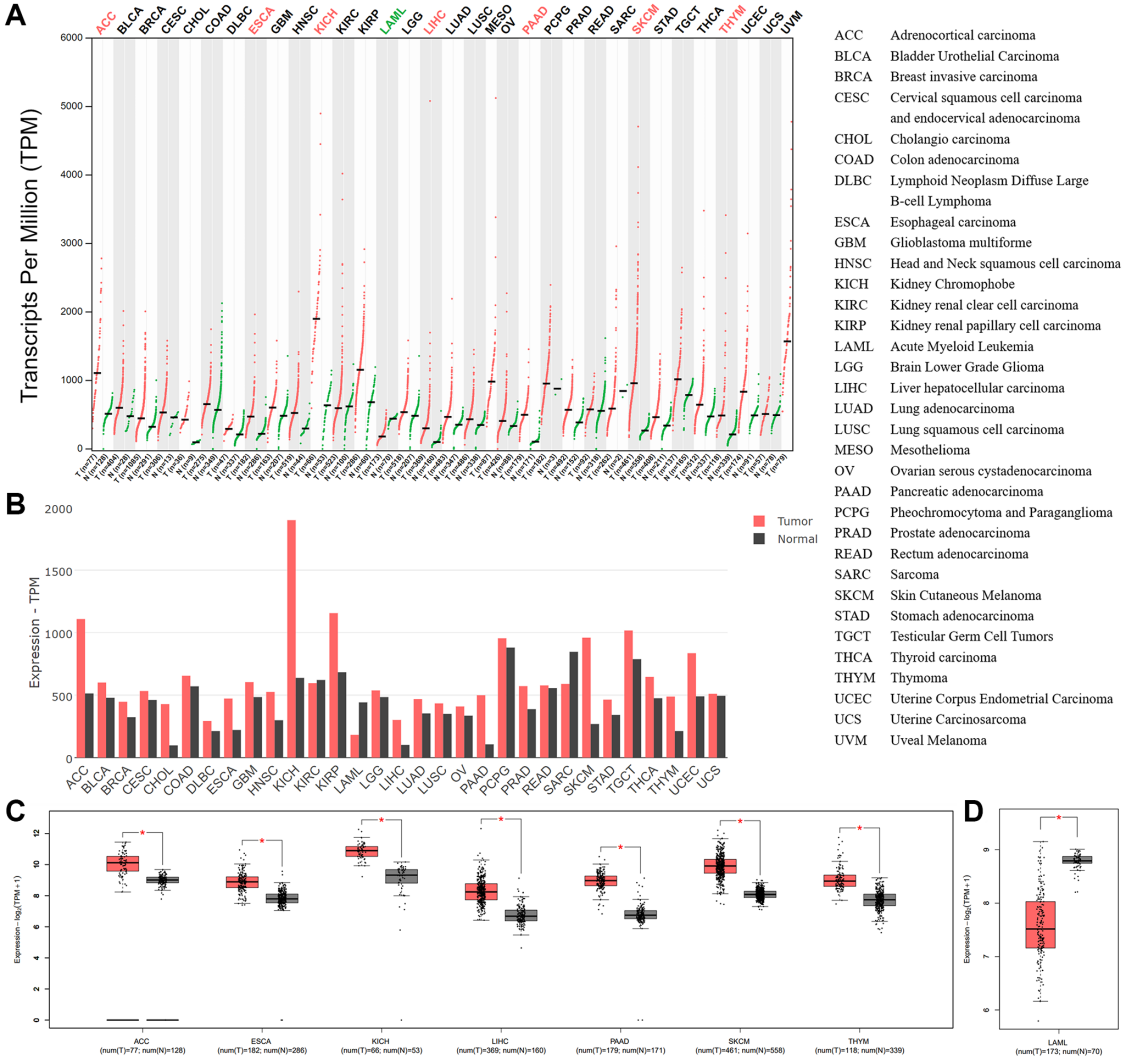
**BSG expressions across all tumor samples and paired normal tissues.** (**A**) BSG expression patterns in cancer tissues and normal tissues (TCGA normal + GTEx normal) by dot plots and (**B**) bar plots. (**C**) BSG expression was upregulated among the seven types of cancer. (**D**) BSG expression was downregulated in one cancer type of LAML. The tumor tissue was colored red, whereas the normal tissue was colored dark gray. Right panel displayed the full name of cancer types. The |log2 (fold change)| cutoff was 1; adjusted *p*-value cutoff was 0.05. ^*^p < 0.05.

To analyze the expression of BSG in pan-cancers, we used a web-based data mining approach based on GEPIA2. The results revealed that BSG mRNA expression in 33 types of tumor tissues and normal tissues was visible in Figure 6A, 6B. BSG mRNA expression level was significantly upregulated in seven cancer types, including ACC, ESCA, KICH, LIHC, PAAD, SKCM and THYM, when compared with corresponding normal tissues (Figure 6C). In addition, BSG expression was significantly downregulated in LAML (Figure 6D). The cutoff of the above analysis was set |Log2FC| as 1 and *p*-value 0.05.

### Immunohistochemical of BSG in lung cancer and normal lung tissues

**Figure 7:**
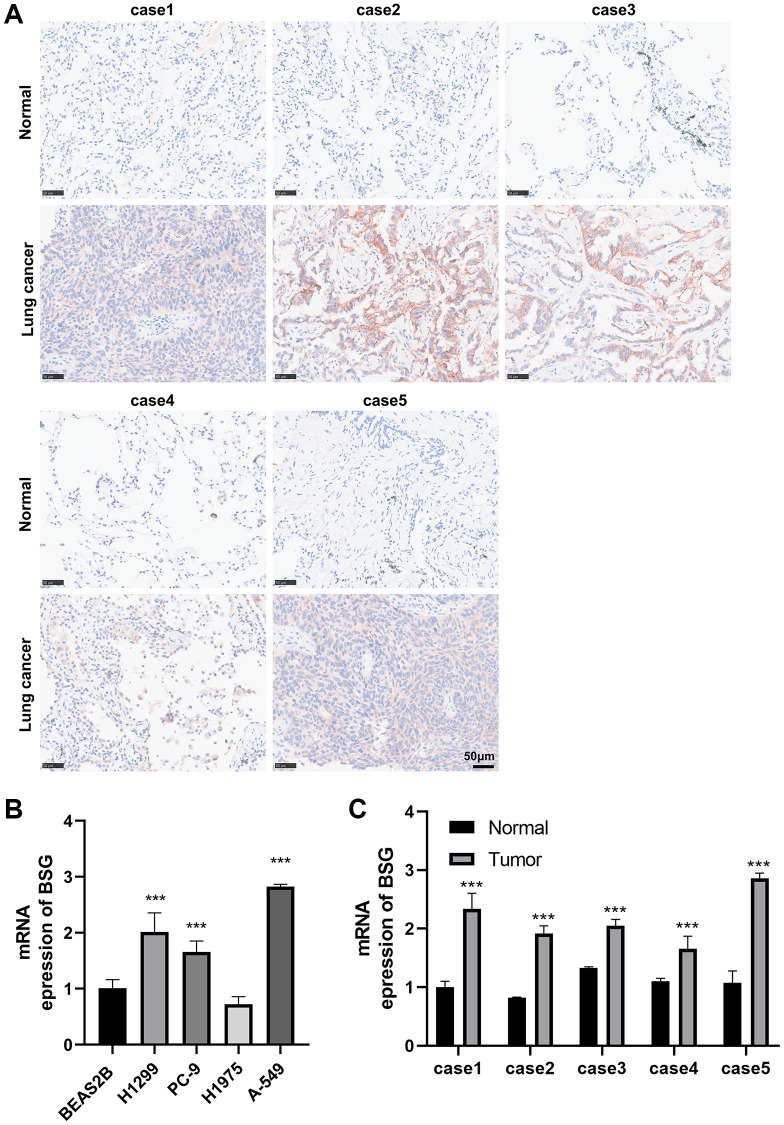
**Immunohistochemical and RT-qPCR analysis of BSG expression.** (**A**) IHC to validate the expression of BSG in five pairs lung cancer tissues. (**B**) qPCR to validate the expression of BSG in lung cancer cell lines. (**C**) qPCR to validate the expression of BSG in five pairs lung cancer tissues. ^***^p < 0.001.

To further explore the protein expression of BSG in lung cancer tissues, we achieved immunohistochemical data from the HPA database. The results indicated that BSG was barely stained in lung normal tissue, but medium-stained in lung squamous cell carcinoma tissue and high-stained in lung adenocarcinoma tissue (Supplementary Figure 1A–1C). To further validate the differential expression of BSG, immunohistochemical was conducted in 5 paired lung cancer tissues and peritumoral lung tissues collected at our institute. The results showed that BSG was highly expressed in lung cancer tissue (Figure 7A).

### Validation of the mRNA expression of BSG in lung cancer tissues and cell lines

We then used real-time PCR to validate the expression of BSG in lung cancer tissues and cell lines. As shown in Figure 7B, compared to normal human lung epithelial cell line (BEAS-2B), BSG was upregulated in most lung cancer cell lines (H1299, PC-9, A-549). Moreover, in five cases of paired cancer and adjacent tissues, the expression of BSG in cancer tissues was significantly higher compared with that in adjacent tissues (Figure 7C).

### Prognostic analysis of BSG in pan-cancers

**Figure 8:**
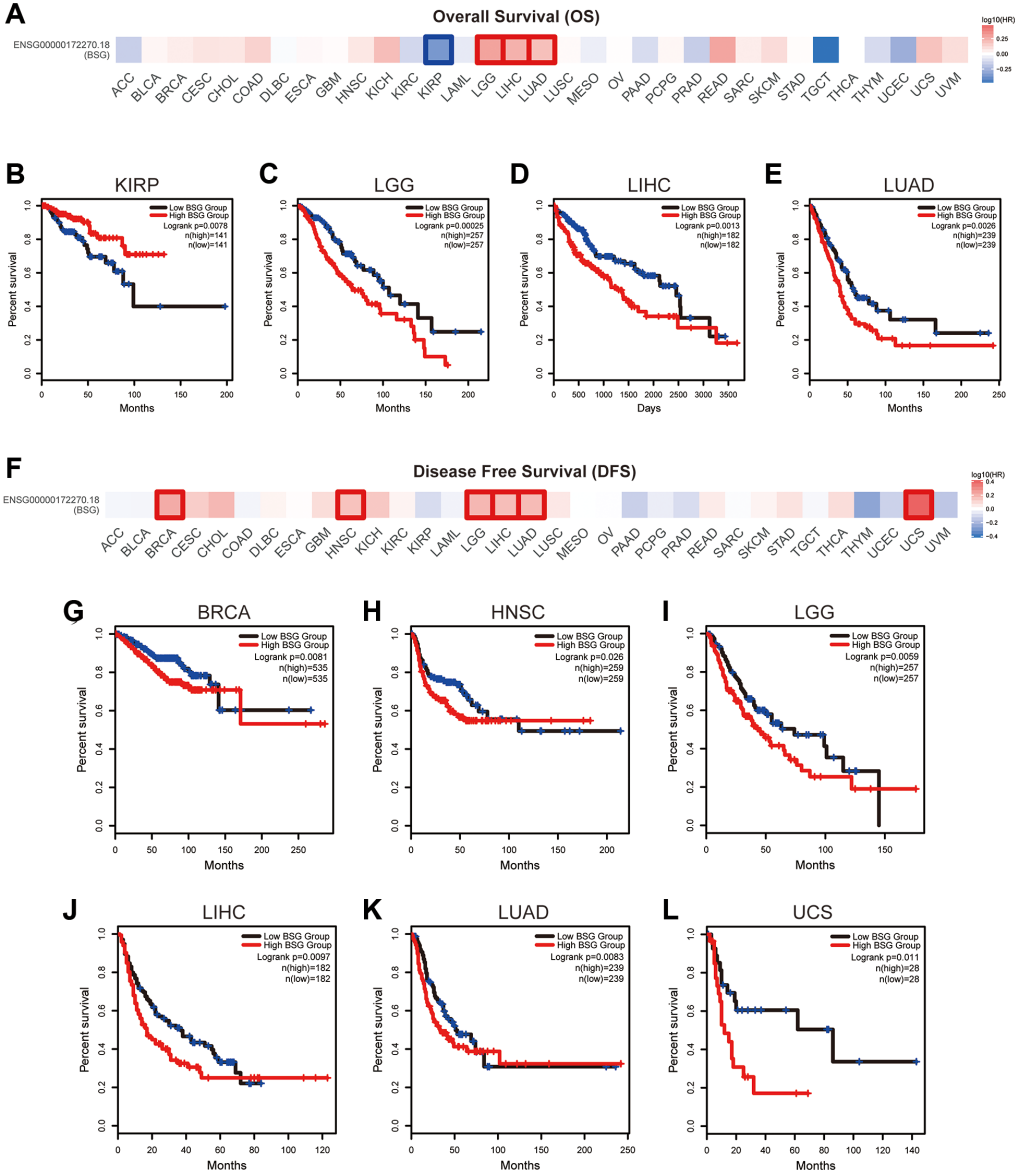
**The prognostic value of BSG across multiple cancer types.** (**A**) Survival heat maps of OS for BSG in 33 TCGA tumor types (the red and blue blocks indicate higher risk and lower risks; the rectangles with frames represent *p* < 0.05). (**B**–**E**) Kaplan–Meier curves of OS for BSG in KIRP, LGG, LIHC and LUAD. (**F**) Survival heat maps of DFS for BSG in 33 TCGA tumor types (the red and blue blocks indicate higher and lower risks; the rectangles with frames represent *p* < 0.05). (**G**–**L**) Kaplan–Meier curves of DFS for BSG in BRCA, HNSC, LGG, LIHC, LUAD and UCS. Abbreviations: OS: overall survival; DFS: disease-free survival. Full names of cancer types were shown in Figure 6.

We conducted Kaplan-Meier analysis to evaluate the association between BSG expression and cancer patients’ prognoses. As shown in Figure 8A–8E, high expression of BSG indicated shorter OS in LGG, LIHC, and LUAD, and longer OS in KIRP. As shown in Figure 8F–8L, high expression of BSG indicated shorter DFS in BRCA, HNSC, LGG, LIHC, LUAD, and UCS. It has been reported that lung cancer patients consistently suffer an increased risk of death from SARS-CoV-2 infection compared to other cancers [51]. Our results showed that in LUAD, BSG was associated with both OS and DFS.

### Comparisons of ACE2, HSPA5, BSG expression levels in pan-cancers and conservation of BSG across species

**Figure 9:**
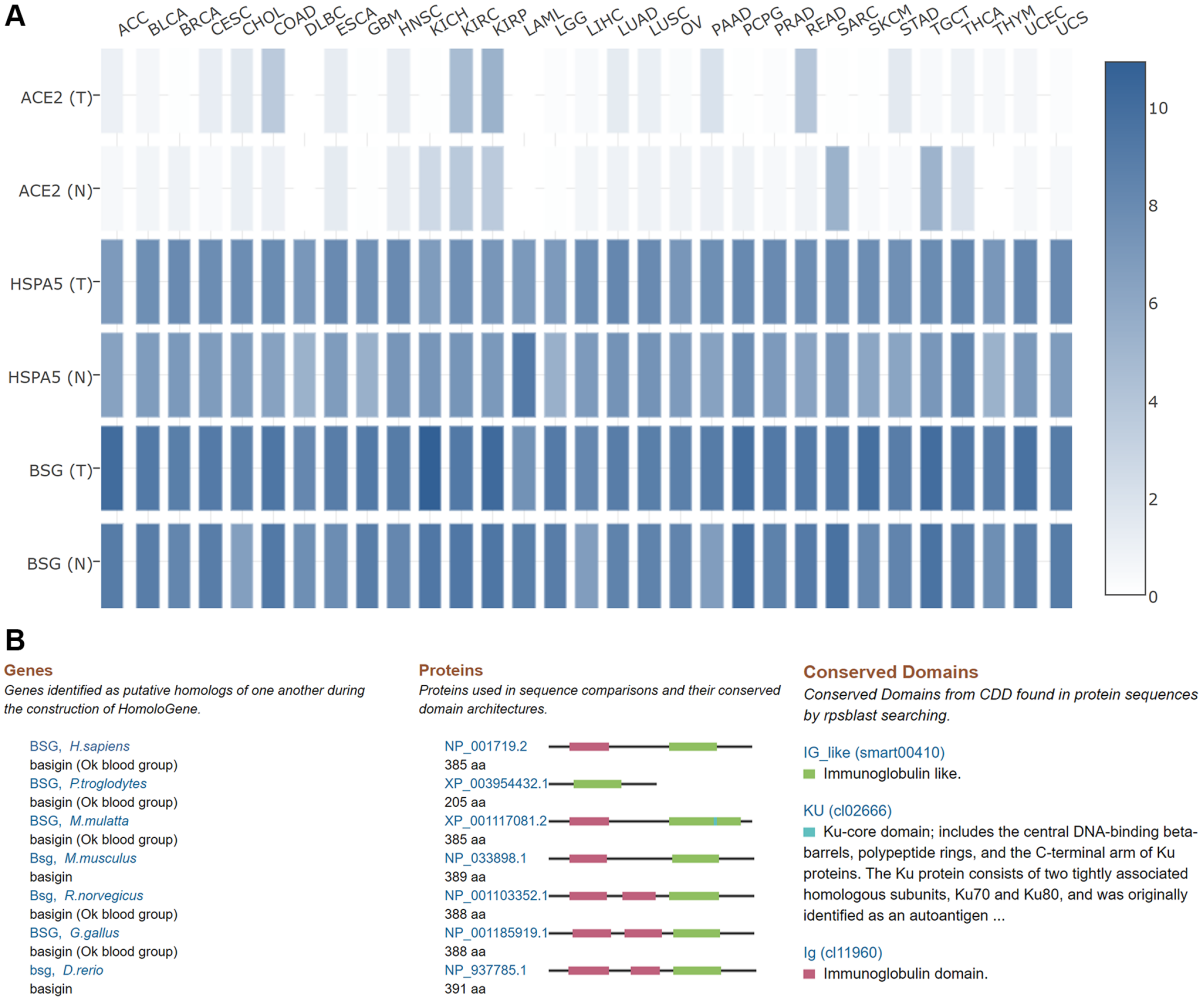
**Comparisons of ACE2, HSPA5 and BSG expression in 31 tumors and their matched normal tissues.** (**A**) In this panel, “T” designates tumor tissues and “N” designates normal tissues. The cancer types were displayed on the top. The density of color in each block represents the median expression value of BSG in tissues, and normalized by the maximum median expression value. (**B**) Homologs and conservations of BSG protein are presented in different species.

It is well known that ACE2, HSP5 and BSG are important entry receptors for SARS-CoV-2 infection [52–54]. Therefore, we compared the expression level of ACE2, HSPA5, BSG in pan-cancers based on TCGA datasets. As shown in Figure 9A, mRNA expression level of BSG was the highest, followed by HSPA5 in most tumor and normal tissues. While, the expression of ACE2 was the lowest. The above results exhibited that BSG might exert important functions for SARS-CoV-2 entry and tumor progression in most of cancer types. The homology and conservation analysis of BSG revealed that BSG is highly conserved in different species, indicating it may also play a role in SARS-CoV-2 entry in other species (Figure 9B).

### Function analysis for co-expressed genes with BSG

**Figure 10:**
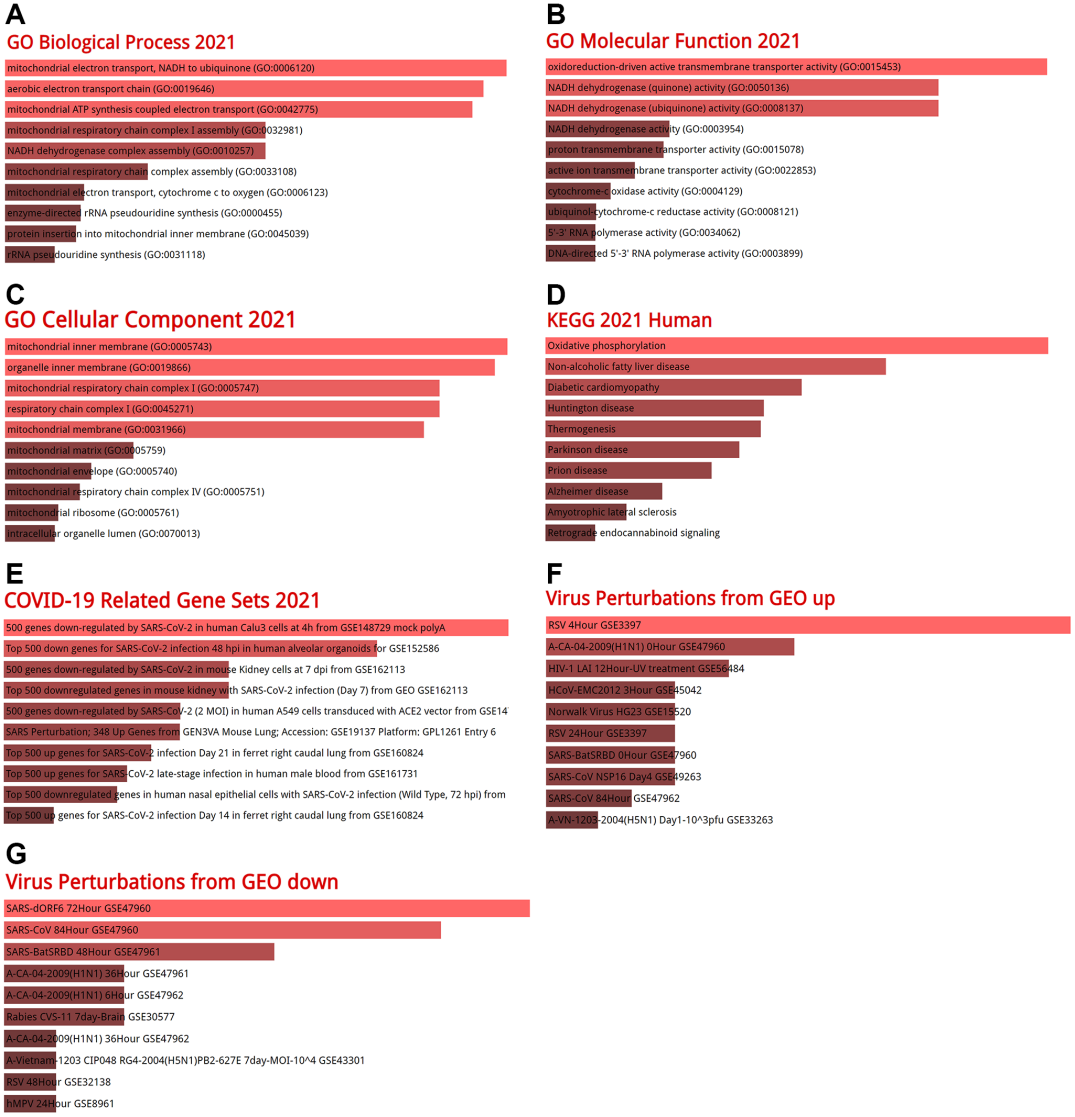
**GO enrichment analysis and KEGG signaling pathway analysis.** (**A**) Biological processes, (**B**) molecular functions, (**C**) cellular components, (**D**) KEGG analysis, (**E**) COVID-19 related gene sets 2021, (**F**) virus perturbations from GEO up, (**G**) virus perturbations from GEO down. Abbreviations: GO: gene ontology; KEGG: Kyoto encyclopedia of genes and genomes.

Co-expression analysis was performed using GEPIA2 and the top 100 genes similar to BSG were identified (Supplementary Table 1). GO terms and KEGG pathways analyses were performed using the Enrichr database. The GO terms contain biological process (BP), molecular function (MF), and cellular component (CC). In BP terms, it was found that the co-expressed genes of BSG significantly participated in mitochondrial electron transport, NADH to ubiquinone (GO: 0006120), aerobic electron transport chain (GO: 0019646), mitochondrial ATP synthesis coupled electron transport (GO: 0042775) (Figure 10A). In MF terms, the co-expressed genes of BSG enriched in oxidoreduction-driven active transmembrane transporter activity (GO: 0015453) and NADH dehydrogenase (quinone) activity (GO: 0050136) (Figure 10B). In CC terms, the co-expressed genes of BSG enriched in mitochondrial inner membrane (GO: 0005743) (Figure 10C). From the KEGG pathway enrichment analysis, the co-expressed genes of BSG enriched in oxidative phosphorylation and non-alcoholic fatty liver disease (Figure 10D). Diseases/drugs enrichment analysis found COVID-19 related gene sets, including 500 genes down-regulated by SARS-CoV-2 in human Calu3 cells at 4 h from GSE148729 mock polyA (Figure 10E). In the virus perturbations from GEO analysis, the co-expressed genes of BSG enriched in RSV 4Hour GSE3397, SARS-dORF6 72Hour GSE47960 and SARS-CoV 84Hour GSE47960 (Figure 10F, 10G). The above results revealed that BSG was mainly enriched in metabolic processes and virus infection.

## DISCUSSION

The COVID-19 epidemic poses an unprecedented challenge to community health and economy. Therefore, it’s significant to explore factors that determine the individual susceptibility and severity to COVID-19. Recently, it has been reported that patients with malignancies were more susceptible to COVID-19 infection and an adverse clinical outcome due to the expression of ACE2, the SARS-CoV-2 receptor [30, 55]. COVID-19 severity varies in different caner types. COVID-19 patients with hematologic cancer are at higher risk of severe outcomes. The reason for this was attributed to decreased myeloid and lymphoid cells, which can cause increased susceptibility to cytokine-mediated inflammation [56–58]. Other COVID-19 patients with lung cancer, melanoma, uterine, and kidney cancer are associated with greater risks [59–64]. Therefore, it’s significant to study the expression level of entry receptors and proteins for the SARS-CoV-2 in various tumor tissues, as the molecular features may influence the susceptibility to SARS-CoV-2 infection. However, ACE2 expression level is very low, especially in lung. Thus, understanding other potential SARS-CoV-2 receptors may provide novel insights and potential therapeutic targets to treat SARS-CoV-2 infection. Our present work emphasized the value of targeting BSG in the treatment of COVID-19 and cancer, and also provided several novel insights for understanding the SARS-CoV-2 pandemic.

At the end of 2020, the interaction of the SARS-CoV-2 spike protein with the host cell receptor BSG was reported for the first time, suggesting that regulation of receptor levels influence the ability of the virus to infect target cells [26]. BSG was related to SARS-CoV-2 infection of immune cells, which do not express ACE2, and served as a novel entry route into human cells [55]. BSG is involved in an indirect interaction between cyclophilin A and viral S proteins. BSG binds directly to the viral S protein, with a remarkable high affinity, followed by dissemination of the virus. [65] Pulmonary edema and vascular permeability in lung disorders are increased by BSG and ACE2, which activates the renin-angiotensin-aldosterone system (RAAS) and contributes to lung destruction [66]. BSG contributes to the COVID-19 symptoms on account of its expression in the inflammatory, infected and tumor cells, and thus forming the basis of the possible COVID-19 therapy [23]. Importantly, an open-label clinical trial of Meplazumab, a humanized therapeutic monoclonal antibody against BSG, which showed significant improvements in patients with COVID-19. Meplazumab blocked BSG interaction with S protein and inhibited host cell infection in a dose-dependent manner [67]. Interestingly, several studies have challenged this concept, and reported no interaction between host cell BSG and recombinant forms of the SARS-CoV-2 spike as well as no changes in the susceptibility to infection of the virus upon knock-out of BSG in lung epithelial cells [65, 68]. Therefore, the role of BSG in SARS-CoV-2 infection remains controversial. Further researches were still needed.

Previous studies showed that BSG played significant roles in tumor progression and may be a biomarker for prognosis. Moreover, BSG is overexpressed in both COVID-19 patients and cancer patients [69, 70]. However, the effects of BSG on SARS-CoV-2 susceptibility and characteristics of patients with malignant tumors in COVID-19 outbreaks are still unclear. It is important to understand the expression of BSG in different normal tissues and malignant tumors.

In this study, using bioinformatic analysis based on online databases, we revealed that BSG was highly conserved in different species and was highly expressed and distributed in gastrointestinal tract, kidney and urinary bladder, and male tissues, which was consistent with ACE2 expression pattern [55]. This co-expression pattern further confirmed that the entry process of SARS-CoV-2 required the cell-surface receptors ACE2 and BSG [71]. High expression of BSG in the digestive system might contribute to the fecal-oral route transmission of SARS-CoV-2. Of note, most patients infected with SARS-CoV-2 also suffer from renal dysfunction [5], which may be due to the high expression of BSG in the kidney and urinary system. Unlike other viruses, the rapid overreaction of the immune system is an important cause of death for the COVID-19 [72]. This difference may suggest the possible involvement of host immune response in the development and maintenance of the pathological changes of COVID-19. What is more, BSG at mRNA expression level was 319.8 folds higher than ACE2 in normal lung tissues, and 151.1 folds in lung cancer tissues. Moreover, the binding affinity of S protein to BSG is 12 folds than that of ACE2 [23]. These evidences indicated an essential role of BSG for SARS-CoV-2 entry in cancer patients through the lungs. And we also observed BSG protein was slightly higher in men than in women. Systematic review analyses of COVID-19 patients with malignancies partially supported these results, that men and patients with lung cancer were more vulnerable to COVID-19 infection [73]. Further comparisons of BSG, HSPA5 and ACE2, all of which are SARS-CoV-2 receptors and cofactors [74–77], we found the expression of BSG to be the highest across almost all kinds of cancer types and normal tissues. All these results suggested an underlying role of BSG in COVID-19 pathogenesis both in normal and cancer tissues. We further analyzed BSG isoform profile and found that the expression level of BSG-003 isoform containing an Ig_3 domain reached the highest level across pan-cancers, suggesting it may be linked with tumor progression and SARS-CoV-2 infection.

To gain more insights into BSG in multiple human cancers, we explored the expression level of BSG and prognostic value across pan-cancers. Differential BSG expression was observed in multiple cancers. BSG mRNA level was significantly upregulated in 7 cancer types, including ACC, ESCA, KICH, LIHC, PAAD, SKCM and THYM, however, was significantly downregulated in LAML. We then used real-time PCR to validate the expression of BSG in lung cancer tissues and cell lines. And we found BSG was upregulated in most lung cancer cell lines (H1299, PC-9, A549). Moreover, in four cases of paired cancer and adjacent tissues, the expression rate of BSG in cancer tissues was significantly higher compared with that in adjacent tissues. The experimental results agree well with bioinformatic analyses. BSG was not only expressed differently in most tumors, but also represented different clinical outcomes in some tumors. High expression of BSG indicated shorter OS in LGG, LIHC, and LUAD, and longer OS in KIRP. Moreover, high expression of BSG indicated shorter DFS in BRCA, HNSC, LGG, LIHC, LUAD and UCS. Our results showed that only in LUAD, BSG was associated with both OS and DFS. These results are similar to previous studies [69, 78–81]. Thus, BSG has the potential to be served as an exciting target for cancer therapy, especially for lung cancer.

Furthermore, our data also demonstrated that in blood cells the BSG was mainly expressed in neutrophils and NK-cells. Previous studies reported that BSG was expressed on various types of proinflammatory cells, such as Th17 cells, neutrophils, NK cells [52, 82], while the key spike protein priming protease, ACE2, is barely expressed [83], which consist with our results. BSG has been shown as a pivotal molecule that mediated host inflammatory and immune responses [20]. Cytokine storm syndrome is the leading cause of mortality in patients with COVID-19. IL-17 is mainly responsible for cytokine storm in SARS-CoV-2, which leads to tissue damage and respiratory failure [84]. SARS-CoV-2 infects the host by binding to BSG and induces Th17 immune response, revealing a critical role of BSG in cytokine storm. Moreover, Meplazumab (BSG antibody) can suppress SARS-COV-2 infection and cytokine storm via preventing direct interactions between BSG and spike protein [29].

The GO and KEGG pathway analyses indicated that BSG was mostly enriched in genes for mitochondria electron transport (GO: 0006120), oxidoreduction-driven active transmembrane transporter activity (GO: 0015453), mitochondrial inner membrane (GO: 0005743) and oxidative phosphorylation. All these pathways are related to cellular metabolism. Previous study confirmed the existence of BSG in human melanoma cell mitochondria. And BSG participated in regulating complex I activities and apoptosis in melanoma via interacting with mitochondrial NDUFS6 [85].

Growing evidence showed that mitochondrial dysfunction was a contributing factor to acute SARS-CoV-2 infection [86]. Moreover, mitochondrial dysfunction was reported to be concerned with the upregulation of ACE2. Since ACE2 is a high affinity binding receptor for SARS-CoV-2, its regulation in correlation with mitochondrial function may have significant clinical implications [87]. Viral infection triggers the release of mitochondrial DNA in host cells and affects mitochondrial dynamics. And the released mitochondrial DNA appears to be involved in regulating the immune reactions and inflammatory response against SARS-CoV-2 infection [88].

However, there are some limitations in our study. Firstly, we are unable to retrieve samples from patients diagnosed with both cancer and COVID-19. Therefore, we are unable to directly determine a significant prognostic effect of BSG expression in these patients. Secondly, the underlying mechanisms of BSG in various cancers need to further experimental exploration.

## CONCLUSIONS

In general, our study analyzed the distribution and expression of a novel SARS-CoV-2 entry BSG in various tissues. We found BSG was expressed in normal tissues and significantly elevated in certain tumor types, suggesting cancer patients are more susceptible to SARS-CoV-2 infection and more likely to develop severe symptoms. Moreover, BSG at mRNA expression level was remarkably higher than ACE2 in normal lung tissues and lung cancer tissues, BSG might be essential for the invasion of SARS-CoV-2 in normal individuals and cancer patients. Overexpression of BSG correlates with poor prognosis in malignant tumors, which suggest that BSG may promote coronavirus infection in patients with malignancies. Our present work emphasized the value of targeting BSG in the treatment of COVID-19 and cancer, and also provided several novel insights for understanding the SARS-CoV-2 pandemic. And future studies are warranted to define the detailed mechanisms.

## Supplementary Materials

Supplementary Figure 1

Supplementary Table 1
