# Supplementary material for: A comprehensive investigation on the receptor BSG expression reveals the potential risk of healthy individuals and cancer patients to 2019-nCoV infection
Source: Aging (Albany NY). 2024 Mar 13;16(6):5412–34. doi: 10.18632/aging.205655 (PMC11006473; doi:10.18632/aging.205655)
Supplement: Supplementary Figure 1 [file aging-16-205655-s001.pdf]

SUPPLEMENTARY FIGURE

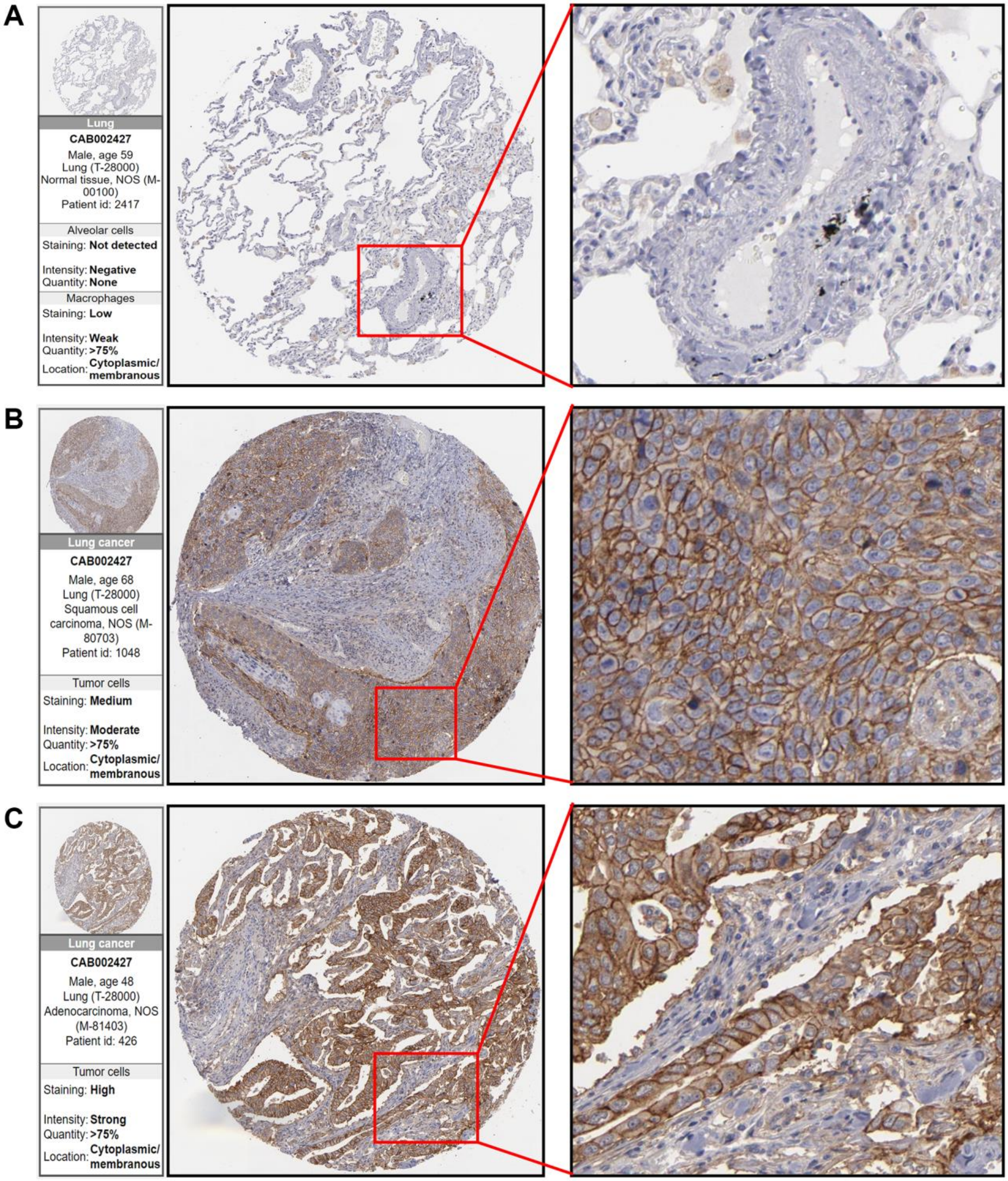

Supplementary Figure 1. Immunohistochemical analysis of BSG in HPA database. Representative staining for BSG in normal lung tissues (**A**), lung squamous cell tissue (**B**) and lung adenocarcinoma tissue (**C**).
