# Supplementary material for: A comprehensive investigation on the receptor BSG expression reveals the potential risk of healthy individuals and cancer patients to 2019-nCoV infection
Source: Aging (Albany NY). 2024 Mar 13;16(6):5412–34. doi: 10.18632/aging.205655 (PMC11006473; doi:10.18632/aging.205655)
Supplement: Supplementary Table 1 [file aging-16-205655-s002.pdf]

SUPPLEMENTARY TABLE

Supplementary Table 1. 100 associated genes for BSG in 33 cancers.

Gene symbol	Gene ID	PCC
NDUFS7	ENSG00000115286.19	0.54
COX4I1	ENSG00000131143.8	0.49
C19orf70	ENSG00000174917.8	0.48
CD320	ENSG00000167775.10	0.48
TEX264	ENSG00000164081.12	0.47
ATP6V1F	ENSG00000128524.4	0.47
NDUFA13	ENSG00000186010.18	0.47
EMC10	ENSG00000161671.16	0.46
NDUFA8	ENSG00000119421.6	0.46
G6PC3	ENSG00000141349.8	0.46
TMEM208	ENSG00000168701.18	0.44
C19orf24	ENSG00000228300.13	0.44
PPP2R1A	ENSG00000105568.17	0.44
MAP2K2	ENSG00000126934.13	0.44
COX7C	ENSG00000127184.10	0.44
RNASEK	ENSG00000219200.10	0.44
LA16c-390E6.5	ENSG00000261641.2	0.43
CD63	ENSG00000135404.11	0.43
C12orf10	ENSG00000139637.13	0.43
NDUFA7	ENSG00000267855.5	0.43
ATP5D	ENSG00000099624.7	0.43
NDUFB10	ENSG00000140990.14	0.43
CTB-25B13.12	ENSG00000267317.2	0.43
TRAPPC2L	ENSG00000167515.10	0.43
POLR2E	ENSG00000099817.11	0.42
ARL2	ENSG00000213465.7	0.42
COX6A1	ENSG00000111775.2	0.42
TUFM	ENSG00000178952.8	0.42
NDUFB2	ENSG00000090266.12	0.42
ILVBL	ENSG00000105135.15	0.41
COX8A	ENSG00000176340.3	0.41
NDUFS3	ENSG00000213619.9	0.41
EIF3C	ENSG00000184110.14	0.41
TRAPPC5	ENSG00000181029.8	0.41
VPS18	ENSG00000104142.10	0.41
NDUFB8	ENSG00000166136.15	0.41
TMED1	ENSG00000099203.6	0.41
CHID1	ENSG00000177830.17	0.41
MRPL28	ENSG00000086504.15	0.41
UQCRC1	ENSG00000010256.10	0.41
SGTA	ENSG00000104969.9	0.4
COMMD4	ENSG00000140365.15	0.4
VEGFB	ENSG00000173511.9	0.4
LRPAP1	ENSG00000163956.10	0.4
HEXA	ENSG00000213614.9	0.4
NDUFA11	ENSG00000174886.12	0.4
C19orf25	ENSG00000119559.15	0.4
VAC14	ENSG00000103043.14	0.4
COQ9	ENSG00000088682.13	0.4
TIMM13	ENSG00000099800.7	0.4
GNPTG	ENSG00000090581.9	0.4
LONP1	ENSG00000196365.11	0.39

CLPP	ENSG00000125656.8	0.39
NDUFA3	ENSG00000170906.15	0.39
WBSCR22	ENSG00000071462.11	0.39
CLN6	ENSG00000128973.11	0.39
ATP5G2	ENSG00000135390.17	0.39
APOO	ENSG00000184831.13	0.39
NDUFA1	ENSG00000125356.6	0.39
PHPT1	ENSG00000054148.17	0.39
VAT1	ENSG00000108828.15	0.39
NDUFC1	ENSG00000109390.11	0.39
HIGD2A	ENSG00000146066.2	0.39
FAM96B	ENSG00000166595.11	0.38
ATP5B	ENSG00000110955.8	0.38
ITFG3	ENSG00000167930.15	0.38
TBL3	ENSG00000183751.14	0.38
WBSCR16	ENSG00000274523.4	0.38
TCEB2	ENSG00000103363.14	0.38
TMEM147	ENSG00000105677.11	0.38
CDC34	ENSG00000099804.8	0.38
PLOD3	ENSG00000106397.11	0.38
MRPL54	ENSG00000183617.4	0.38
GADD45GIP1	ENSG00000179271.2	0.38
SCYL1	ENSG00000142186.16	0.38
FKBP2	ENSG00000173486.12	0.38
COPE	ENSG00000105669.12	0.38
MRPL34	ENSG00000130312.6	0.38
PRKCSH	ENSG00000130175.9	0.37
UQCR11	ENSG00000127540.11	0.37
CUTA	ENSG00000112514.15	0.37
FAM195A	ENSG00000172366.19	0.37
NDUFA2	ENSG00000131495.8	0.37
POLRMT	ENSG00000099821.13	0.37
HAGHL	ENSG00000103253.17	0.37
SLC4A2	ENSG00000164889.12	0.37
IDH3G	ENSG00000067829.18	0.37
RENBP	ENSG00000102032.12	0.37
MRPS24	ENSG00000062582.13	0.37
STUB1	ENSG00000103266.10	0.37
RPUSD2	ENSG00000166133.17	0.37
ATP6V0C	ENSG00000185883.10	0.37
SLC25A11	ENSG00000108528.13	0.37
NDUFB7	ENSG00000099795.6	0.37
NDUFS8	ENSG00000110717.10	0.37
TIMM22	ENSG00000177370.4	0.37
TSR3	ENSG00000007520.3	0.37
MRPL17	ENSG00000158042.8	0.36
SLC25A3	ENSG00000075415.12	0.36
POLR2J	ENSG00000005075.15	0.36
